# Treatment of Sprains

**Published:** 1886-05

**Authors:** 


**Treatment of Sprains.**—The proper treatment of sprains varies with the severity of the injury. In mild cases a cure can frequently be obtained in a day or two, by immersing the affected limb in cold water for a few minutes, and then applying an ordinary roller bandage, and keeping the limb elevated and at rest until the pain and swelling subside. In some cases better results can be obtained by placing the injured part in hot water instead of cold, and then applying the bandage. In other cases, more benefit will result from the alternate applications of hot and cold water. Lotions of lead-water and laudanum, or vinegar and water will also be found serviceable at times.

In severe cases the bowels should be freely opened by saline cathartics, and the pain and constitutional excitement controlled by the administration of morphia and aconite, or morphia and gelsemium. Locally, the greatest benefit can de obtained in these cases from the use of cold applications during the first twenty- four hours. The limb should be elevated, and icebags or cold compresses placed on the affected surface. Evaporating lotions also may be employed with advantage.

At the expiration of twenty-four hours light massage should be resorted to, in order to hasten the removal of the effused fluids. The manipulations should be conducted with extreme gentleness, and should not extend over a period of more than five minutes at first. After massage has been employed for two or three days the limb should be placed in a plaster bandage and the patient permitted to walk with the aid of a cane. In some cases the plaster bandage may be applied on the second day with the certainty of a speedy urce.—*Medical Bulletin.*

**Information Wanted.**—We all hear of, read about, and even sometimes see children marked by their mothers. While some of these markings are not plain, here is a case, well or badly done, just as the reader takcs it:

Mrs. B. D., living near K., Minn., while between four and five months pregnant, saw a dog attack a pig in the yard, the dog tearing off completely the pig's left ear. Four months later Mrs. B. gave birth to a daughter, now fourteen years old and presenting the following anomalies of the left side of the face: Complete absence of lobe of the ear, there being only a small mole posterior to meatus; meatus normal and hearing good. Complete paralysis of facialis sinister, with, of course, the usual symptoms. Complete paralysis of abducens oculi, hence permanent strabismus. The part of cornea not covered by eyelid during sleep, the seat of keratitis. Speech normal. A C. AMUNDSON, M. D.

